# Dynamic evolution of flavor profiles and antioxidant capacities in black rice tea during continuous roasting

**DOI:** 10.3389/fnut.2026.1879933

**Published:** 2026-07-02

**Authors:** Weiping Zhang, Yueling Xu, Jiani Liu, Xinsheng Li, Hongxing Zheng, Wengang Jin, A. M. Abd El-Aty

**Affiliations:** 1 School of Biological Science and Engineering, Shaanxi University of Technology, Hanzhong, China; 2 Shaanxi Black Organic Food Engineering Center, Shaanxi University of Technology, Hanzhong, China; 3 Department of Pharmacology, Faculty of Veterinary Medicine, Cairo University, Giza, Egypt; 4 Department of Medical Pharmacology, Faculty of Medicine, Atatürk University, Erzurum, Türkiye

**Keywords:** black rice tea, E-nose, gas chromatography–ion mobility spectrometry (GC–IMS), roasting time, volatile organic compounds

## Abstract

In this study, the dynamic evolution of functional components and volatile flavor profiles in black rice tea across different roasting times (90 °C, 0, 5, 10, 20, and 30 min) was investigated. Physicochemical assays, electronic nose, and gas chromatography–ion mobility spectrometry (GC–IMS) were employed to characterize the quality transformation mechanisms. The results indicated that while total flavonoids decreased continuously during heating, total anthocyanins reached a maximum at 20 min. The overall antioxidant capacity remained stable and was likely maintained by the compensatory generation of Maillard reaction products. E-nose analysis revealed an expansion of volatile aromas accompanied by a continuous depletion of water-soluble taste substrates. GC–IMS identified 46 volatile organic compounds and revealed a distinct sequential transition: initial raw-odor alcohols dissipated rapidly within the first 5 min; aliphatic aldehydes and ketones accumulated from 10 to 20 min via lipid oxidation; and roast-associated heterocyclic compounds, particularly 2-pentylfuran, peaked at 20 min through advanced Maillard reactions. Principal component analysis (PCA) confirmed the clear differentiation of these roasting stages. Overall, roasting for 20 min was recommended to achieve optimal quality of black rice tea. These results may provide certain references for quality control of black rice tea during roasting in the future.

## Introduction

Grain-based substitute teas have gained popularity because of their unique sensory characteristics and potential health benefits (1). The antioxidant capacity of black rice (*Oryza sativa* L.) is widely recognized, which is attributed primarily to functional components such as anthocyanins and flavonoids (2, 3). Roasting is an essential processing step in the production of black rice tea (4). During thermal treatment, chemical reactions such as the Maillard reaction and the degradation of native precursors occur (5). Meanwhile, lipid oxidation during thermal processing is also very important for roasted grains. These heat-induced changes not only reduce the raw odor of unroasted grains but also generate volatile organic compounds (VOCs) that contribute to the characteristic roasted flavor of the tea (6).

Although previous studies have investigated the nutritional composition and final volatile profiles of various grain teas (7), most of these evaluations focus on end products rather than intermediate processing stages. During roasting, the consumption of nonvolatile precursors and the generation of volatile compounds occur simultaneously (8). Moderate heating promotes the development of a roasted aroma and the release of internal active substances; conversely, prolonged thermal exposure can induce the degradation of heat-sensitive antioxidants and the formation of off-flavors (9). However, the time-dependent evolution of these flavor profiles and their correlation with fluctuations in functional components under continuous thermal stress remain insufficiently characterized (10). Clarifying this dynamic transition is necessary for optimizing processing parameters and ensuring the overall quality of the tea.

Therefore, the objective of this study was to evaluate the impact of roasting durations on the functional components and flavor profiles of black rice tea. Firstly, raw black rice was pretreated (washed, drained, and dried to a moisture content of 14%) and roasted at 90 °C for various durations (0, 5, 10, 20, and 30 min). Changes in total anthocyanins, total flavonoids, and antioxidant capacities (ABTS and DPPH assays) were continuously monitored. Concurrently, electronic nose combined with gas chromatography–ion mobility spectrometry (GC–IMS) were employed to characterize the odor shifts and volatile evolution. This work will provide a theoretical basis for understanding flavor formation and optimizing the roasting process of black rice tea.

## Materials and methods

### Materials and reagents

Black rice *(Oryza sativa* L., cv. Heiguan) was obtained from Yangxian County, Hanzhong city, Shaanxi Province, China. Chemical reagents, including 2, 2-diphenyl-1-picrylhydrazyl (DPPH), 22′-azino-bis (3-ethylbenzothiazoline-6-sulfonic acid) (ABTS), rutin (purity >98%, analytical grade), and cyanidin-3-glucoside (purity >98%, analytical grade), were purchased from Sigma–Aldrich (St. Louis, MO, USA). Analytical grade solvents, including ethanol and hydrochloric acid, were obtained from Sinopharm Chemical Reagent Co., Ltd. (Shanghai, China). A mixture of n-ketones (C4–C9) used for the retention index (RI) calculation was secured from Aladdin Biochemical Technology Co., Ltd. (Shanghai, China).

### Preparation of black rice tea

The raw black rice was subsequently washed with distilled water, drained, and dried in a hot air dryer at 50 °C until the moisture content reached 14%. The dried grains were then roasted in a pan at constant 90 °C. On the basis of preliminary screening, samples were collected at five specific roasting durations: 0, 5, 10, 20, and 30 min. The unroasted sample (0 min) was used as the control. After roasting, all the samples were immediately cooled to room temperature and vacuum-sealed in aluminum foil bags for further analysis.

### Determination of total anthocyanins, flavonoids, and antioxidant capacities

The total anthocyanins content (TAC) was determined using the pH differential method (measuring wavelength were 520 nm and 700 nm, respectively) and expressed as milligrams of cyanidin-3-glucoside equivalents per gram of sample (mg/g) (11). The calculation formula was as follows (Equation 1): *A* = (*A*_520_−*A*_700_)*pH*_1.0_−(*A*_520_−*A*_700_)*pH*_4.5_


$$\begin{array}{l} TAC\left(mg/g\right)=\frac{A\times MW\times DF\times 1000}{\varepsilon \times l} \end{array}$$ (1)

Where A was absorbance, MW was molecular weight (449.2 g/mol) of cyanidin-3-glucoside. DF was dilution factor. εwas molar absorptivity (26,900 g·mol^−^^1^·cm^−1^) of cyanidin-3-glucoside.

The total flavonoid content was measured by an aluminum chloride colorimetric assay and expressed as milligrams of rutin equivalents per gram of sample (mg/g) (12). The antioxidant capacities were evaluated using ABTS and DPPH radical scavenging assays according to the protocols described previously (13). All the absorbance values were recorded using a UV-1800 spectrophotometer (Shimadzu, Kyoto, Japan).

### Sensory evaluation

The sensory profiles of the black rice tea were evaluated by a panel of 20 assessors trained before providing sensory scores according to standard evaluation protocols (14). The tea infusion was prepared by brewing 10 g of the sample in 150 mL of boiling water. The mixture was covered for 1 min to assess the initial aroma, followed by color observation at 5 min and subsequent taste evaluation. The comprehensive sensory score was calculated on the basis of five weighted attributes: appearance (15%), liquor color (10%), aroma (30%), taste (35%), and infused grains (10%). To ensure objectivity, consensus descriptive profiles and acceptable scores were generated through panel means.

### E-nose

An electronic nose system (Supernose, ISENSO, France) equipped with a 14-channel metal–oxide semiconductor gas sensor array (S1–S14) was utilized to evaluate the overall volatile profiles. In accordance with the sensing characteristics (15, 16), the 14 sensors were categorized into broad target classes: sensors S1, S8, S12, S13, and S14 are sensitive primarily to alkanes and combustible gases; S2, S6, S7, and S9 respond to alcohols, aldehydes, ketones, and aromatic compounds; S4 and S11 detect sulfides; S5 is responsive to nitrogen oxides; and S3 detects ozone; and S10 responds to hydrogen.

### GC–IMS analysis

Volatile organic compounds (VOCs) were identified using a FlavorSpec^®^ GC–IMS system (G.A.S., Dortmund, Germany) equipped with a CTC-PAL 3 autosampler (17). Briefly, 2.0 g of sample was placed in a 20 mL headspace vial and incubated at 60 °C for 15 min at 500 rpm. Afterward, a 500 μL headspace volume was injected into the system (injector temperature of 85 °C) in splitless mode. Chromatographic separation was performed on an MXT-5 capillary column (15 m × 0.53 mm, 1.0 μm; Restek, USA) maintained at 60 °C. Nitrogen (purity ≥99.999%) was used as the carrier gas, with a programmed flow rate of 2.0 mL/min for 2 min, which was increased to 10.0 ml/min over 8 min, after which it was finally increased to 100.0 ml/min over 10 min. The drift tube was operated at 45 °C under a constant nitrogen flow of 150 ml/min. The ionization source was tritium (3H). Retention indices (RIs) were calculated using n-ketones (C4–C9) as external references. Data processing, including the generation of topographic plots, gallery plots, and principal component analysis (PCA), was performed using VOCal software (version 0.4.03, G.A.S.).

### Statistical analysis

All experiments were conducted in triplicate, and the results are expressed as the mean ± standard deviation. The data were subjected to one-way analysis of variance (ANOVA), and significant differences (*p* < 0.05) were determined using Duncan's multiple range test in SPSS 26.0 (IBM, Chicago, IL, USA). Data visualization and PCA plots were generated using GraphPad Prism 9.0 and R software (version 4.2.1).

## Results

### Physicochemical properties and antioxidant capacities

**Figure 1:**
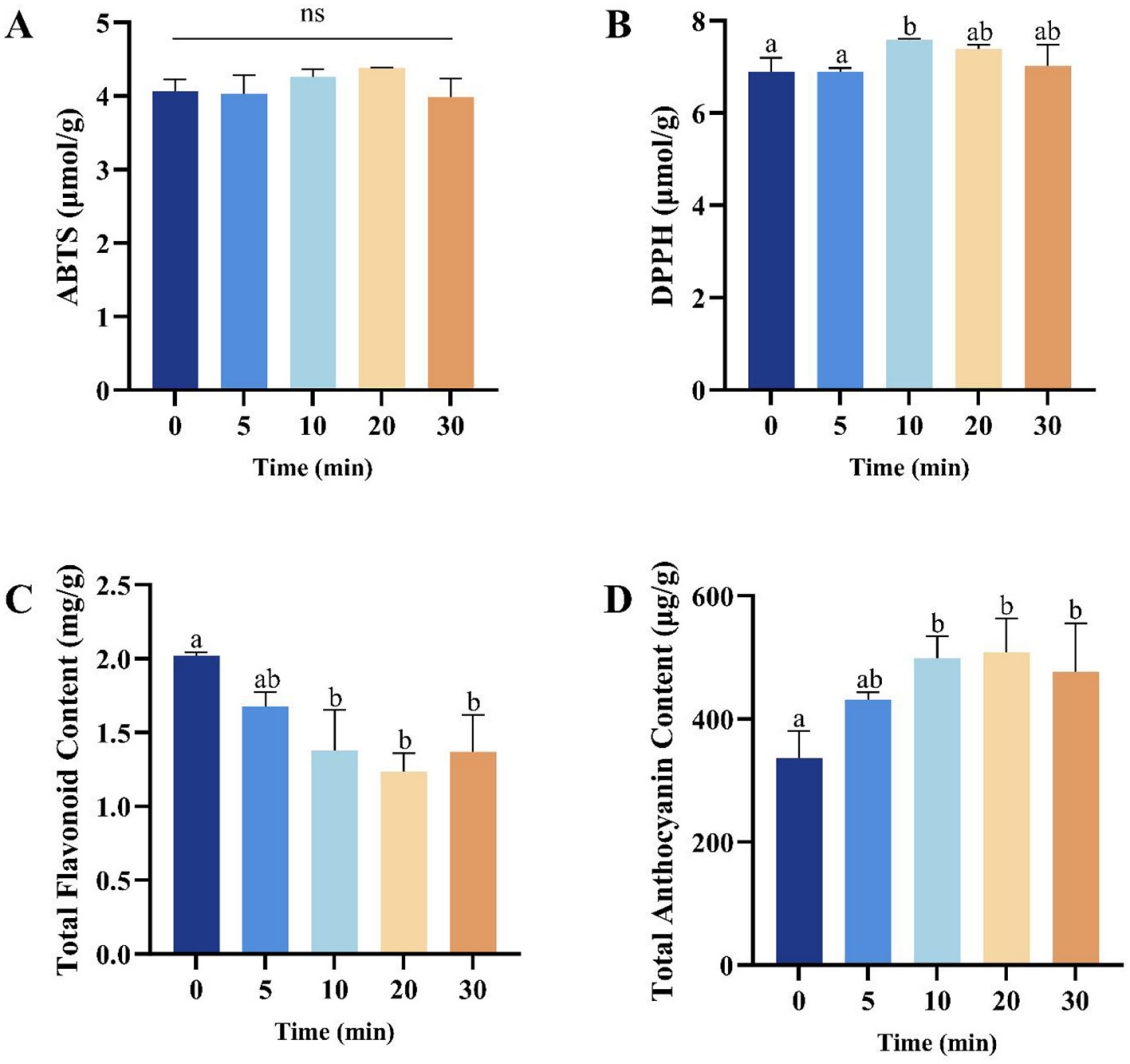
Effects of different roasting times (0, 5, 10, 20, and 30 min) on physicochemical properties and antioxidant capacities of black rice tea. **(A)** ABTS radical scavenging activity. **(B)** DPPH radical scavenging activity. **(C)** Total flavonoids content. **(D)** Total anthocyanins content. The data are expressed as the mean ± standard deviation (*n* = 3). Different lowercase letters above the bars indicate significant differences (*p* < 0.05) according to Duncan's multiple range test.

The dynamic changes in the physicochemical components and antioxidant capacities of black rice tea after different roasting times are illustrated in Figure 1. As shown in Figure 1C, the total flavonoids content continuously and significantly decreased (*p* < 0.05) with prolonged roasting, dropping from its maximum at 0 min to lower levels at 20–30 min. Conversely, the total anthocyanins content (Figure 1D) initially increased, reaching a peak value at 10–20 min, followed by a slight decrease at 30 min.

In terms of antioxidant capacity, the ABTS radical scavenging activity (Figure 1A) remained relatively stable and did not significantly differ (ns, *p* > 0.05) across all roasting times. The DPPH radical scavenging activity (Figure 1B) increased significantly during the initial heating stage, peaking at 10 min, and remained high throughout the subsequent roasting process.

### Appearance and sensory evaluation

**Figure 2:**
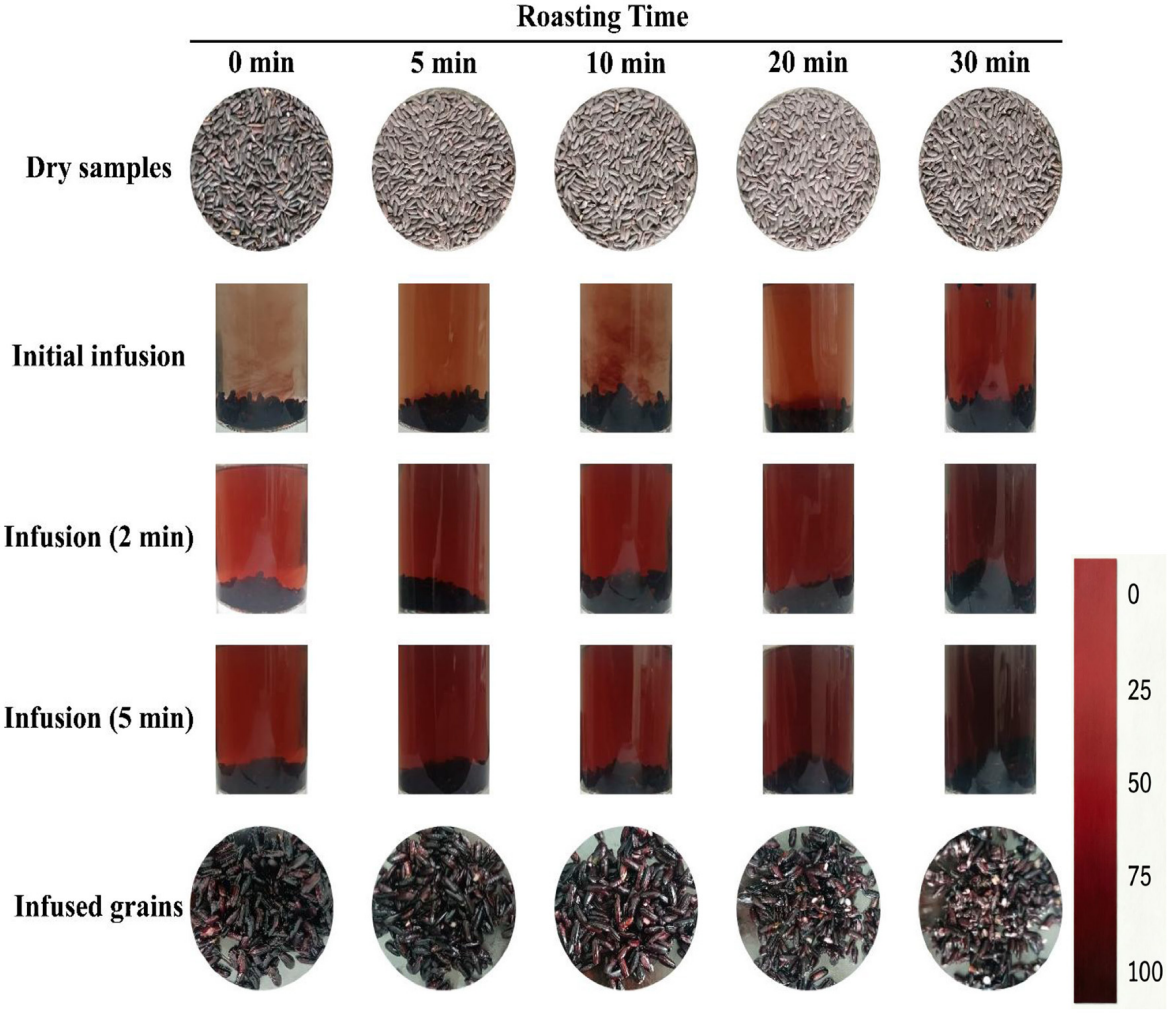
Visual appearance of black rice tea processed at different roasting times. The images display the macroscopic morphological changes of the dry roasted grains, the corresponding tea infusions, and the infused grains after brewing.

The visual characteristics of dry samples, infused grains, and tea infusions at different stages are presented in Figure 2. As the roasting time increased, the appearance of the dry samples shifted from tight and glossy to slightly cracked surface, while the color of the tea infusions deepened significantly from clear light red (0 min) to turbid dark red (30 min).

The consensus sensory descriptive profiles are summarized in Table 1. The unroasted sample (0 min) was characterized by a faint rice aroma and a bland taste. With moderate roasting (10–20 min), the sensory attributes improved significantly. The 20 min sample exhibited the most favorable profile, characterized by a rich roasted aroma and a “sweet, mellow, and thick” taste. However, prolonged roasting at 30 min resulted in an intense burnt aroma and a slightly astringent taste, leading to a decrease in overall acceptability.

**Table 1:** Consensus sensory descriptive profiles and comprehensive sensory evaluation scores of black rice tea at different roasting times.

Roasting time (min)	Appearance	Liquor color	Aroma	Taste	Infused grains	Consensus score range
0	Black and glossy, tight	Clear, light red	Faint rice aroma	Bland	Hard and intact	60–70
5	Black and glossy, tight	Slightly bright, light red	Moderate rice aroma	Relatively thick	Hard and slightly swollen	70–80
10	Black and glossy, slightly loose	Slightly turbid, brownish-red	Rich rice aroma	Relatively mellow	Slightly soft and swollen	80–85
20	Black, slightly wrinkled surface	Slightly turbid, amber-red	Rich roasted and sweet rice aroma	Sweet, mellow, thick with a sweet aftertaste	Soft, swollen, and slightly cracked	85–95
30	Black, cracked surface	Turbid, dark red	Intense burnt rice aroma	Mellow but slightly astringent	Swollen and cracked	75–85

### E-nose evaluation of flavor profiles

**Figure 3:**
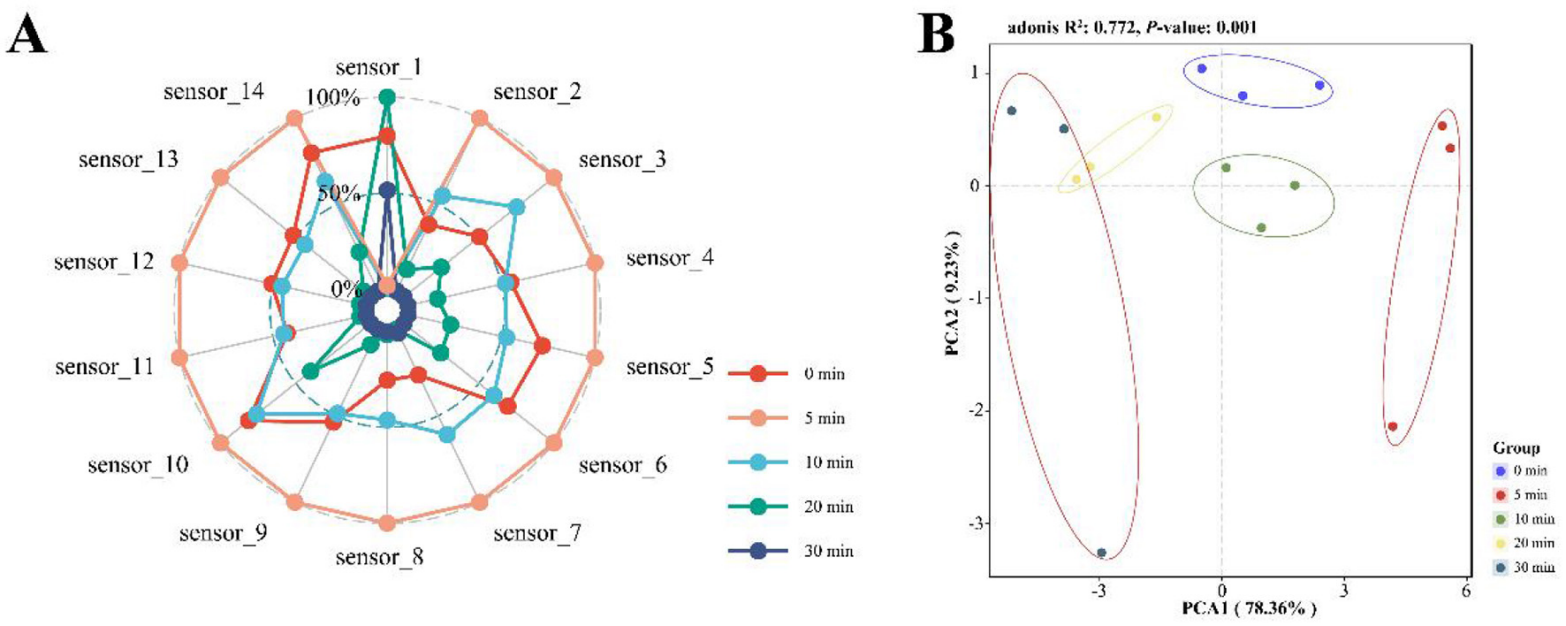
E-nose evaluation of black rice tea at different roasting times. **(A)** Normalized radar chart of the electronic nose (E-nose) sensor responses; **(B)** principal component analysis (PCA) score plot based on the E-nose response data.

Macroscopic flavor shifts were monitored using an electronic nose (E-nose) (Figure 3). The E-nose normalized radar chart (Figure 3A) revealed a sharp expansion in sensor responses at 5 min, indicating a rapid increase in volatile compounds during the early stage of roasting. The subsequent E-nose PCA plot (Figure 3B) clearly revealed spatial separation among the five groups (*p* = 0.001), with the aroma profiles migrating continuously along the first principal component (PC1) from 0 to 30 min.

### Volatile organic compounds (VOCs) by GC–IMS

**Figure 4:**
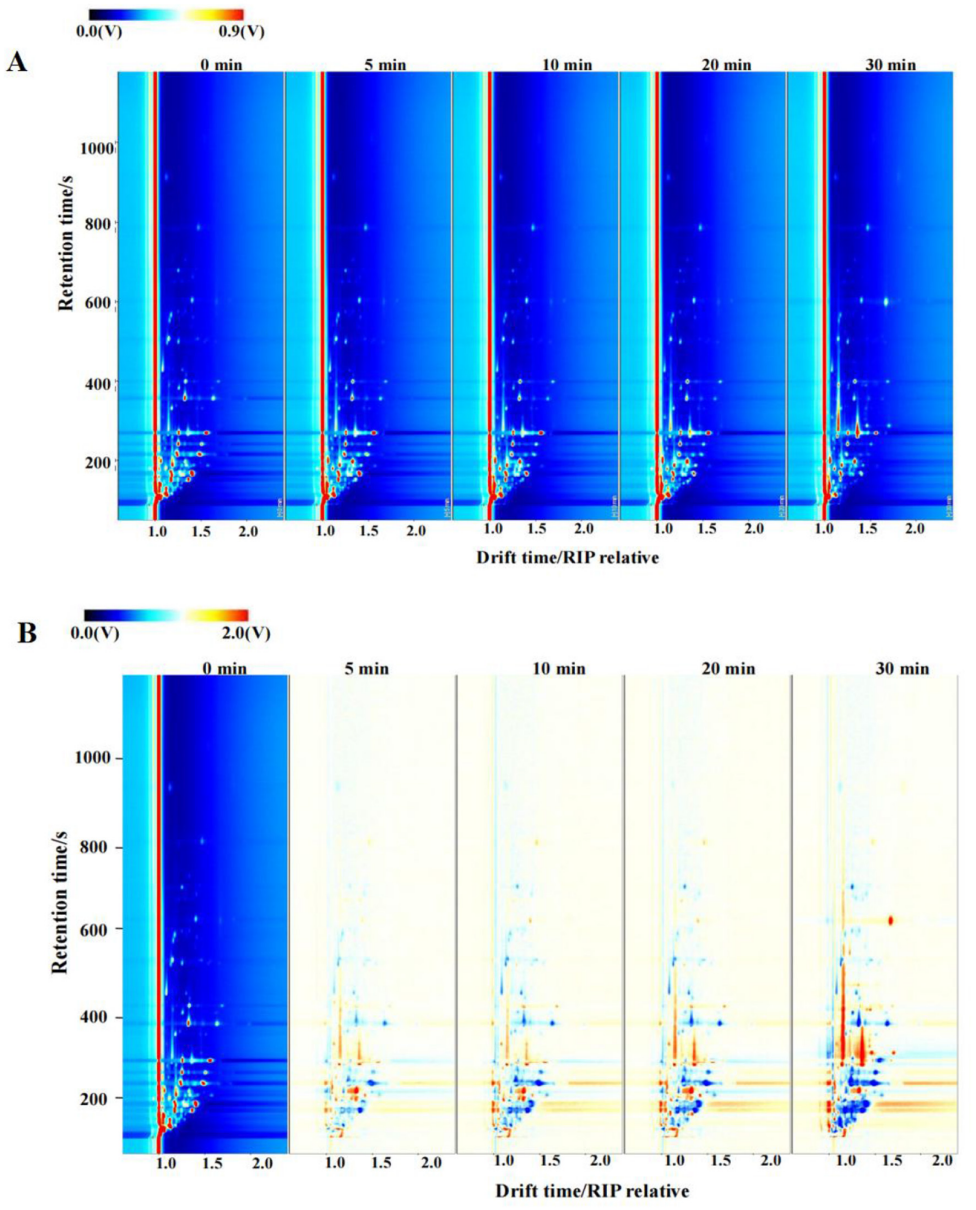
Global topography and difference plots of volatile organic compounds in black rice tea after different roasting times. **(A)** Global two-dimensional (2D) topographic plot; **(B)** difference topographic plot using the unroasted sample (0 min) as the reference. In the difference plot, red dots indicate compound concentrations higher than the reference concentration, whereas blue dots indicate concentrations lower than the reference concentration.

To identify the specific volatile compounds responsible for the aroma variations, GC–IMS was employed. The topographic plot and difference plot (Figures 4A, B) provided a global visualization of the VOCs. Using the 0 min sample as a reference, a large number of red spots emerged and intensified in the 5–30 min samples (Figure 4B), confirming the massive generation of new volatile substances induced by roasting.

**Figure 5:**
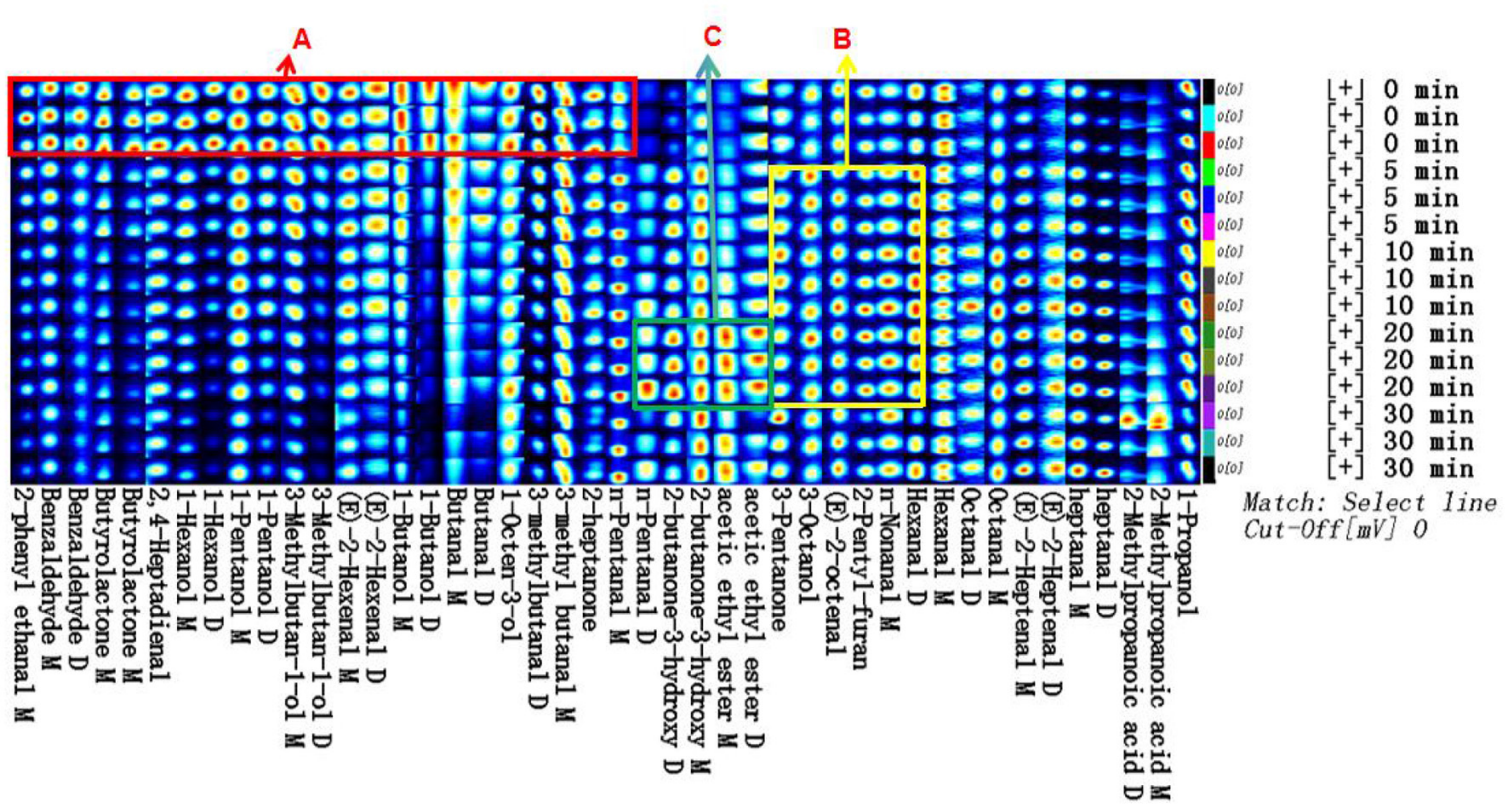
Gallery plot (fingerprint) of the 46 identified VOCs in black rice tea at different roasting times. Each row represents a sample replicate, and each column represents the signal intensity of a specific volatile compound.

A total of 46 volatile signal peaks were identified, and the GC-IMS qualitative profile of volatile components of black rice tea during different roasting times (0, 5, 10, 20, and 30 min) were shown in Supplementary Figure S1. And their time-dependent evolution were visualized in the gallery plot (Figure 5). The fingerprint clearly divided the VOCs into three evolution patterns. Compounds associated with raw or green odors [e.g., hexanol, 1-octen-3-ol, and (E)-2-hexenal] were highly abundant at 0 min but dissipated rapidly after heating (Region A in Figure 5). Roast-associated compounds (e.g., 2-pentylfuran, hexanal, and nonanal) increased significantly at 5–20 min, dominating the aroma profile (Region B in Figure 5). At 20–30 min, compounds such as pentanal, 3-hydroxy-2-butanone, and acetic ethyl ester accumulated (Table1 and Figure 5), contributing to the mellow and sweet background notes (Region C in Figure 5).

**Figure 6:**
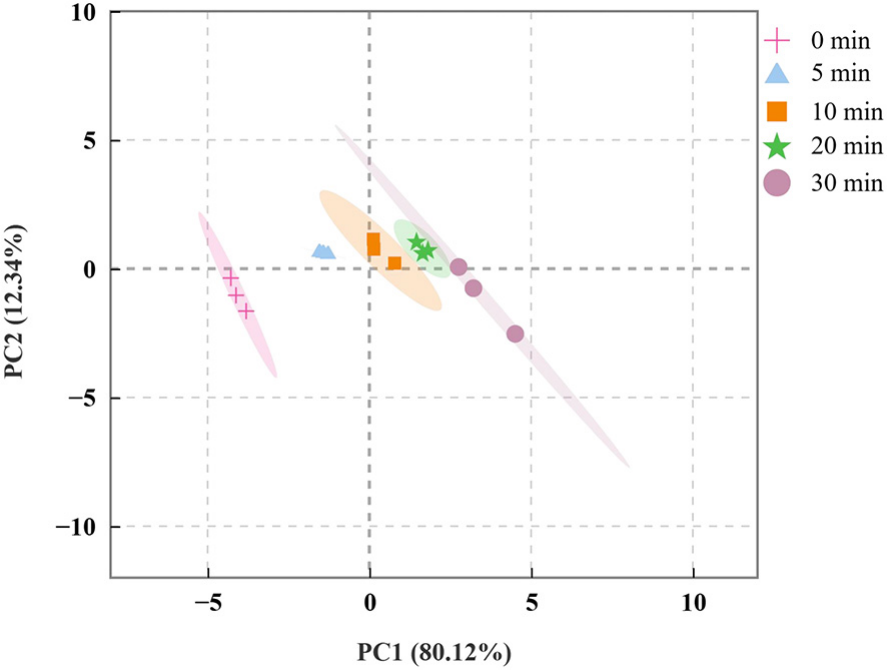
Principal component analysis (PCA) score plot based on the signal intensities of the volatile organic compounds identified by GC–IMS for black rice tea at different roasting times.

Finally, a PCA based on the GC–IMS signal intensities was conducted (Figure 6). The first two principal components accounted for 92.46% of the total variance (PC1 = 80.12%, PC2 = 12.34%). The PCA score plot exhibited a distinct trajectory without overlap, perfectly distinguishing the black rice tea samples at different roasting stages.

## Discussion

Flavonoids and total anthocyanins are major secondary metabolites that govern the antioxidant capacity of pigmented rice during thermal processing (18). In this study, total flavonoids decreased continuously, whereas total anthocyanins peaked at 20 min. This was accompanied by stable ABTS and DPPH scavenging capacities (Figure 1). Yamuangmorn et al. (19) reported that moderate roasting (e.g., 100 °C for 20 min) temporarily enhances the extractability of anthocyanins before their ultimate thermal degradation. Furthermore, Arora et al. (20) reported the retention of overall antioxidant capacity in roasted black rice despite significant anthocyanin loss, attributed to the formation of heat-induced antioxidative compounds. Several reports also proved that there were correlation between total flavonoids, anthocyanins and antioxidant activities (21–24). Therefore, the stable antioxidant activity of our black rice tea is probably maintained by a compensatory mechanism via multiple contributions (flavonoids, anthocyanins, and Maillard products) during roasting.

Visual appearance and sensory scores are practical metrics for evaluating physical changes and consumer acceptability of grain beverages. As roasting progressed to 20 min, the black rice grains developed slight surface cracks and yielded a dark red infusion, resulting in the highest sensory score for the roasted aroma and taste (Figure 2 and Table 1). He et al. (21) reported that roasting significantly increased the browning index of germinated brown rice, which directly correlated with the development of key flavor substances. Similarly, Wang et al. (25) reported maximum sensory acceptability for colored highland barley at a moderate roasting stage, as this provides an optimal balance between chemical release and visual appeal. The optimal sensory quality of the 20 min black rice tea is presumably due to the ideal balance between pigment extraction and flavor accumulation.

Electronic nose allow for the simultaneous tracking of volatile aromas and water-soluble taste substrates (26). The E-nose responses expanded and achieved clear PCA separation (Figure 3). Wang et al. (27) demonstrated that combined E-nose and E-tongue systems effectively capture this dynamic difference between aroma generation and taste degradation in heated rice matrices. Park et al. (28) found that the aroma was diverse during rice bran roasting, but the sweetness decreased because of the thermal degradation of taste precursors, which was similar with present results. The sensory shifts observed in our samples are thus governed by the ongoing conversion of nonvolatile taste precursors into volatile odorants via thermal reactions.

GC–IMS visualizes the overall shifts in volatile profiles, particularly the loss of raw odors during early heating stages. The topographic and gallery plots revealed that green-odor alcohols dissipated rapidly after 5 min, accompanied by the emergence of new volatile signals (Figures 4, 5). Wang et al. (29) highlighted the efficiency of GC–IMS in monitoring these global flavor transitions across different degrees of roasting of rice. Specifically, Jin et al. (30) reported the rapid dissipation of initial alcohols in colored grains, indicating that heat disrupts the grain matrix and accelerates the volatilization of low-boiling-point compounds. The elimination of raw odors in our black rice tea is likely driven by this initial physical volatilization and the onset of thermal degradation at the beginning of roasting (20, 21).

Straight-chain aldehydes and ketones are crucial intermediate volatiles that contribute preliminary fatty and floral notes to roasted grain infusions (31). The gallery plot revealed that aliphatic compounds, such as nonanal and hexanal, accumulated steadily between 10 and 20 min of roasting (Figure 5). Peng et al. (32) reported that specific thermal treatments promote the generation of these aliphatic aldehydes and ketones, shifting the overall volatile profile of pseudocereal matrices. Li et al. (33) also reported a continuous accumulation of hexanal and nonanal, attributing their formation primarily to the thermal degradation and oxidation of lipids. The enrichment of these aliphatic compounds in our samples is consistently driven by the thermal oxidation of internal lipids and Maillard reaction during prolonged roasting (21, 33).

Heterocyclic compounds and esters serve as critical markers for the signature roasted flavor, enabling statistical differentiation of processing stages (34). Roast-associated volatiles, such as 2-pentylfuran and acetic ethyl ester, peaked at 20 min (Figure 5F) and clearly separated the roasting groups in the PCA model (Figure 6). Zhang et al. (35) reported that tracking these specific trace volatiles via GC–IMS provides a reliable foundation for the multivariate differentiation of thermally processed foods. Similarly, Wang et al. (29) attributed the late-stage formation of furans and pyrazines in roasted barley to advanced thermal reactions, which facilitated clear PCA clustering. The distinct roasted aroma and clear PCA separation of our 20 min samples are probably determined by lipid oxidation and Maillard reaction. This study used a single black rice cultivar (Heiguan) and one roasting temperature. Generalizability to other cultivars or roasting conditions is unknown and needed for its direct application.

## Conclusion

In conclusion, the roasting duration can affect the nutritional quality and volatile flavor profiles of black rice tea. Continuous roasting led to a continuous decrease in total flavonoids, but a specific roasting duration of 20 min yielded maximum total anthocyanins extractability and antioxidant activities. The combination of E-nose and GC–IMS analyses elucidated a distinct volatile flavor profiles of black rice tea during roasting. Overall, the roasting duration of 20 min at 90 °C was recommended to achieve optimal quality for preparing black rice tea. However, the precise quality characteristics and flavor traits during roasting should be performed in the future.

## Data Availability

The original contributions presented in the study are included in the article/supplementary material, further inquiries can be directed to the corresponding author.

## References

[1] Xiong Y Zhang P Warner RD Shen S Fang Z Shen S . Cereal grain-based functional beverages: from cereal grain bioactive phytochemicals to beverage processing technologies, health benefits and product features. Crit Rev Food Sci Nutr. (2022) 62:2404–2431. doi: 10.1080/10408398.2020.1853037 33938780

[2] Wu L Zhai M Yao Y Dong C Shuang S Ren G. Changes in nutritional constituents, anthocyanins, and volatile compounds during the processing of black rice tea. Food Sci Biotechnol. (2013) 22:917–923. doi: 10.1007/s10068-013-0164-z

[3] Das S Kumari T Babu SNC Kumar S Deka SC. Bioactive compounds, functional properties, health benefits, and food applications of black rice: a comprehensive review. Food Chem Adv. (2025) 7:917–923. doi: 10.1016/j.focha.2025.101028

[4] Cañizares L Meza S Peres B Rodrigues L Jappe SN Coradi PC . Functional foods from black rice (*Oryza sativa* L.): an overview of the influence of drying, storage, and processing on bioactive molecules and health-promoting effects. Foods. (2024) 13:1088. doi: 10.3390/foods13071088 38611392 PMC11011668

[5] Hong B Zhang S Yuan D Shan S Zhang JY Sha DX . Changes in bioactive constituents in black rice metabolites under different processing treatments. Foods. (2025) 14:1630. doi: 10.3390/foods14091630 40361712 PMC12071922

[6] Xiong Y Zhang P Luo J Johnson S Fang Z. Effect of processing on the phenolic contents, antioxidant activity and volatile compounds of sorghum grain tea. J Cereal Sci. (2019) 85:6–14. doi: 10.1016/j.jcs.2018.10.012 31617213

[7] Sun H Wang P Zhang P Ajlouni S Fang Z. Changes in phenolic content, antioxidant activity, and volatile compounds during processing of fermented sorghum grain tea. Cereal Chem. (2020) 97:612–25. doi: 10.1002/cche.10277

[8] Gu Z Jin Z Schwarz P Rao J Chen B. Unveiling the dynamic response of volatile development during barley malt roasting via untargeted and pseudo-targeted flavoromics: a time course study. Food Chem. (2025) 468:142477. doi: 10.1016/j.foodchem.2024.142477 39706122

[9] Gui A Gao S Zheng P Feng Z Liu P Ye F . Dynamic changes in non-volatile components during steamed green tea manufacturing based on widely targeted metabolomic analysis. Foods. (2023) 12:1551. doi: 10.3390/foods12071551 37048372 PMC10094149

[10] Xu W Zhu Y Lin L Tunyaluk B Li P. Dynamic changes in volatile components during dark tea wine processing. LWT. (2024) 194:142477. doi: 10.1016/j.lwt.2024.115783

[11] Lee J Durst RW Wrolstad RE. Determination of total monomeric anthocyanin pigment content of fruit juices, beverages, natural colorants, and wines by the pH differential method: collaborative study. J AOAC Int. (2019) 88:1269–78. doi: 10.1093/jaoac/88.5.1269 16385975

[12] Shraim AM Ahmed TA Rahman MM Hijji YM. Determination of total flavonoid content by aluminum chloride assay: a critical evaluation. LWT. (2021) 150:1269–1278. doi: 10.1016/j.lwt.2021.111932

[13] Jakubczyk K Melkis K Maciejewska-Markiewicz D Muzykiewicz-Szymańska A Nowak A Skonieczna-Żydecka K. Innovative approaches to enhancing kombucha through flavour additives: a phytochemical and antioxidant analysis. Food Funct. (2025) 16:1442–57. doi: 10.1039/D4FO05135A 39898619

[14] Zhang H Wang J Wang H Cheng C Zhang X Xue J . Comparative analysis of asparagus tea processing and flavor component analysis. LWT. (2024) 194:1442–57. doi: 10.1016/j.lwt.2024.115795

[15] Fu L Yang G Liu L Ma Y Zhang X Zhang X . Analysis of volatile components of Auricularia auricula from different origins by GC-MS combined with electronic nose. J Food Qual. (2020) 2020:8858093. doi: 10.1155/2020/8858093

[16] Beullens K Mészáros P Vermeir S Kirsanov D Legin A Buysens S . Analysis of tomato taste using two types of electronic tongues. Sens Actuators B Chem. (2008) 131:10–7. doi: 10.1016/j.snb.2007.12.024

[17] Xiao Y Huang Y Chen M Zhu C He C Li Z . Characteristic fingerprints and change of volatile organic compounds of dark teas during solid-state fermentation with *Eurotium cristatum* by using HS-GC-IMS, HS-SPME-GC-MS, E-nose and sensory evaluation. LWT. (2022) 169:113925. doi: 10.1016/j.lwt.2022.113925

[18] Mbanjo EGN Kretzschmar T Jones H Ereful N Blanchard C Boyd LA . The genetic basis and nutritional benefits of pigmented rice grain. Front Genet. (2020) 11:229. doi: 10.3389/fgene.2020.00229 32231689 PMC7083195

[19] Yamuangmorn S Sreethong T Saenchai C Rerkasem B Prom-U-Thai C. Effects of roasting conditions on anthocyanin, total phenolic content, and antioxidant capacity in pigmented and non-pigmented rice varieties. Int Food Res J. (2021) 28:73–82. doi: 10.47836/ifrj.28.1.07

[20] Arora S Virdi IK Sharanagat VS Kheto A Dhua S Suhag R . Roasting of black rice (*Oryza sativa* L.): change in physico-functional, thermo-pasting, antioxidant and anthocyanin content. J Food Meas Charact. (2021) 15:2240–50. doi: 10.1007/s11694-021-00828-7

[21] He M Guo T Li D Xie C Wang P Yang R. Effects of roasting on physicochemical characteristics and flavor substances of germinated brown rice. Food Sci Biotechnol. (2024) 34:125–35. doi: 10.1007/s10068-024-01655-4 39758724 PMC11695666

[22] Zhao CR Chen Q Ma X Yang TY. The chemical composition of the walnut pellicle and its benefits to health. Food Med Homol. (2024) 1:9420007. doi: 10.26599/FMH.2024.9420007

[23] Zhao DN Zhou XM Gong XJ Quan WX. Optimization of ultrasound-assisted extraction of flavonoids from *Emilia prenanthoidea* DC. using response surface methodology and exploration of the ecological factors on total flavonoid and antioxidant activity. Food Med Homol. (2024) 1:9420017. doi: 10.26599/FMH.2024.9420017

[24] Sun-Waterhouse DX Chen XY Liu ZH Waterhouse GIN. Transformation from traditional medicine-food homology to modern food-medicine homology. Food Med Homol. (2024) 1:9420014. doi: 10.26599/FMH.2024.9420014

[25] Wang C Zhang Z Zhang X Tian X Chen K Zeng X. Characterization of volatile compounds by HS-GC-IMS and chemical composition analysis of colored highland barley roasted at different temperatures. Foods. (2022) 11:2921. doi: 10.3390/foods11182921 36141048 PMC9498828

[26] Tan J Xu J. Applications of electronic nose (e-nose) and electronic tongue (e-tongue) in food quality-related properties determination: a review. Artif Intell Agric. (2020) 4:104–15. doi: 10.1016/j.aiia.2020.06.003

[27] Wang T Yang L Xiong Y Wu B Liu Y Qiao M . Characterization of flavor profile of steamed beef with rice flour using gas chromatography-ion mobility spectrometry combined with intelligent sensory (electronic nose and tongue). Front Nutr. (2024) 11:1435364. doi: 10.3389/fnut.2024.1435364 39229587 PMC11368871

[28] Park H Ban Y Hong SJ Yoon S Moon HS Yu SY . Antioxidant and chemosensory properties of rice (*Oryza sativa* L.) bran under different oven-roasting conditions. Food Chem. (2025) 476:143496. doi: 10.1016/j.foodchem.2025.143496 39987805

[29] Wang Z-Y Dou B-X Ma C-M Zhang Y-L Liu Y Zhang N. Evaluation of volatile flavor compounds in rice with different degrees of roasting based on GC-IMS and PCA analysis. Food Sci Technol. (2022) 43:100222. doi: 10.1590/fst.100222

[30] Jin W Zhao S Sun H Pei J Gao R Jiang P. Characterization and discrimination of flavor volatiles of different colored wheat grains after cooking based on GC-IMS and chemometrics. Curr Res Food Sci. (2023) 7:100222. doi: 10.1016/j.crfs.2023.100583 PMC10484957 37691695

[31] Wang S Chen H Sun B. Recent progress in food flavor analysis using gas chromatography–ion mobility spectrometry (GC–IMS). Food Chem. (2020) 315:100222. doi: 10.1016/j.foodchem.2019.126158 32014672

[32] Peng S Li Y Liu H Tuo Y Dang W Wang W . Influence of germination and roasting on the characteristic volatile organic compounds of quinoa using sensory evaluation, E-nose, HS-GC-IMS, and HS-SPME-GC-MS. Food Chem X. (2024) 22:100222. doi: 10.1016/j.fochx.2024.101441 38756471 PMC11096820

[33] Li M Sun M Ren W Man L Chai W Liu G . Characterization of volatile compounds in donkey meat by gas chromatography–ion mobility spectrometry (GC–IMS) combined with chemometrics. Food Sci Anim Resour. (2024) 44:165–77. doi: 10.5851/kosfa.2023.e67 38229857 PMC10789554

[34] Shakoor A Zhang C Xie J Yang X. Maillard reaction chemistry in formation of critical intermediates and flavour compounds and their antioxidant properties. Food Chem. (2022) 393:100222. doi: 10.1016/j.foodchem.2022.133416 35696950

[35] Zhang L Shi P Sun J Xie M Wang H Shi T . Analysis of roasted peanuts based on GC–MS combined with GC–IMS. Food Sci Nutr. (2024) 12:1888–901. doi: 10.1002/fsn3.3882 38455194 PMC10916660

