# Supplementary material for: Dynamic evolution of flavor profiles and antioxidant capacities in black rice tea during continuous roasting
Source: Front Nutr. 2026 Jul 2;13:1879933. doi: 10.3389/fnut.2026.1879933 (PMC13373877; doi:10.3389/fnut.2026.1879933)
Supplement: Supplementary file 1 [file Supplementary_file_1.docx]

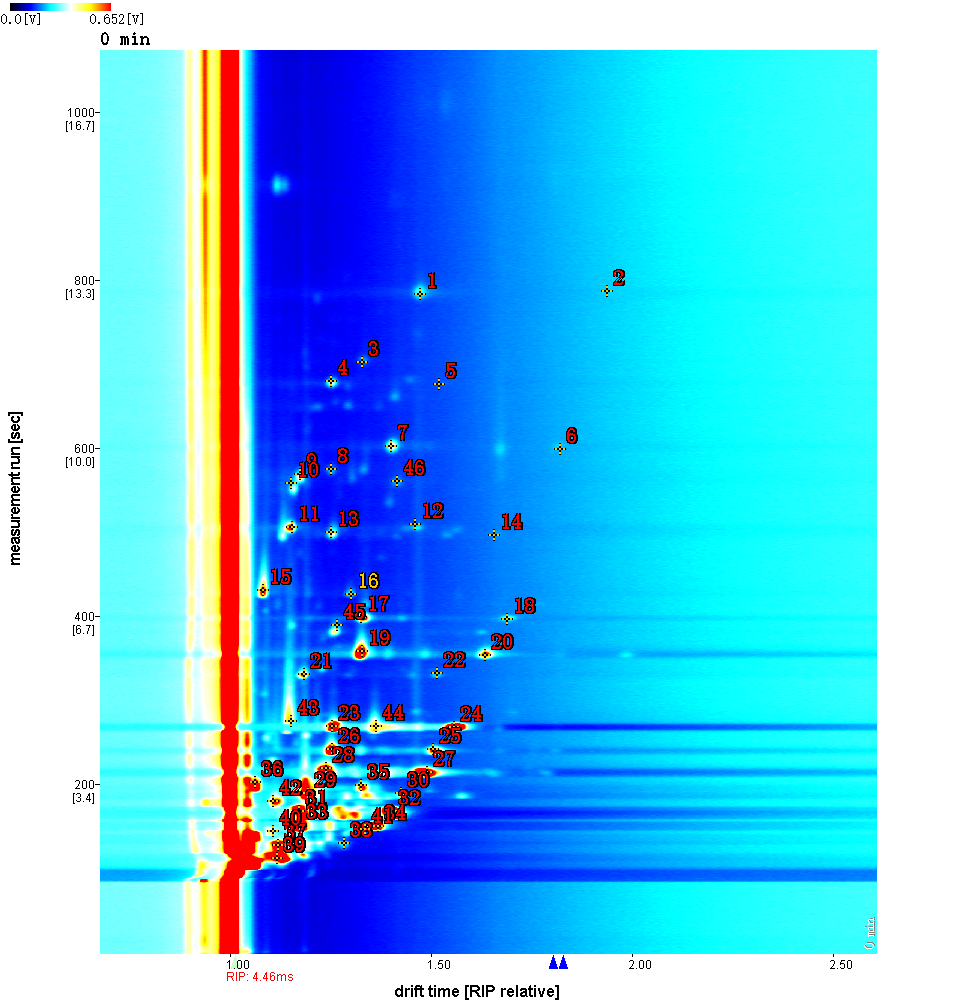

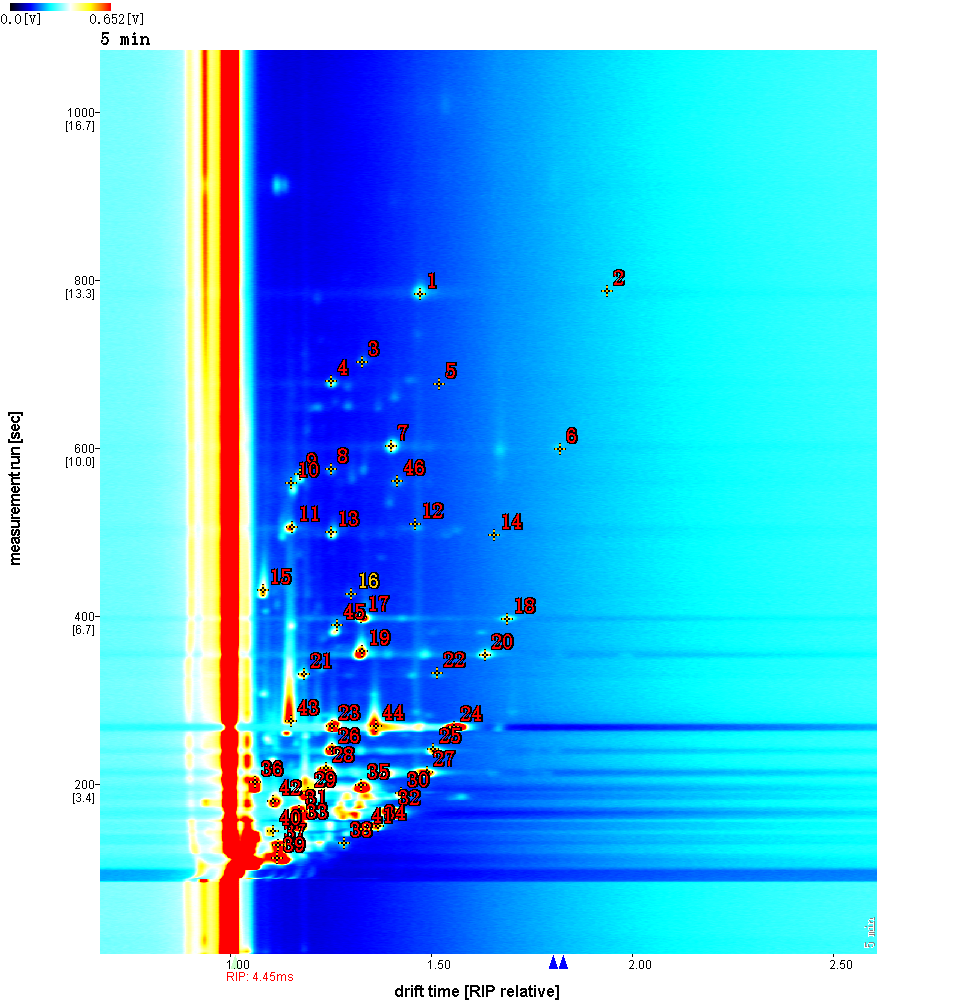

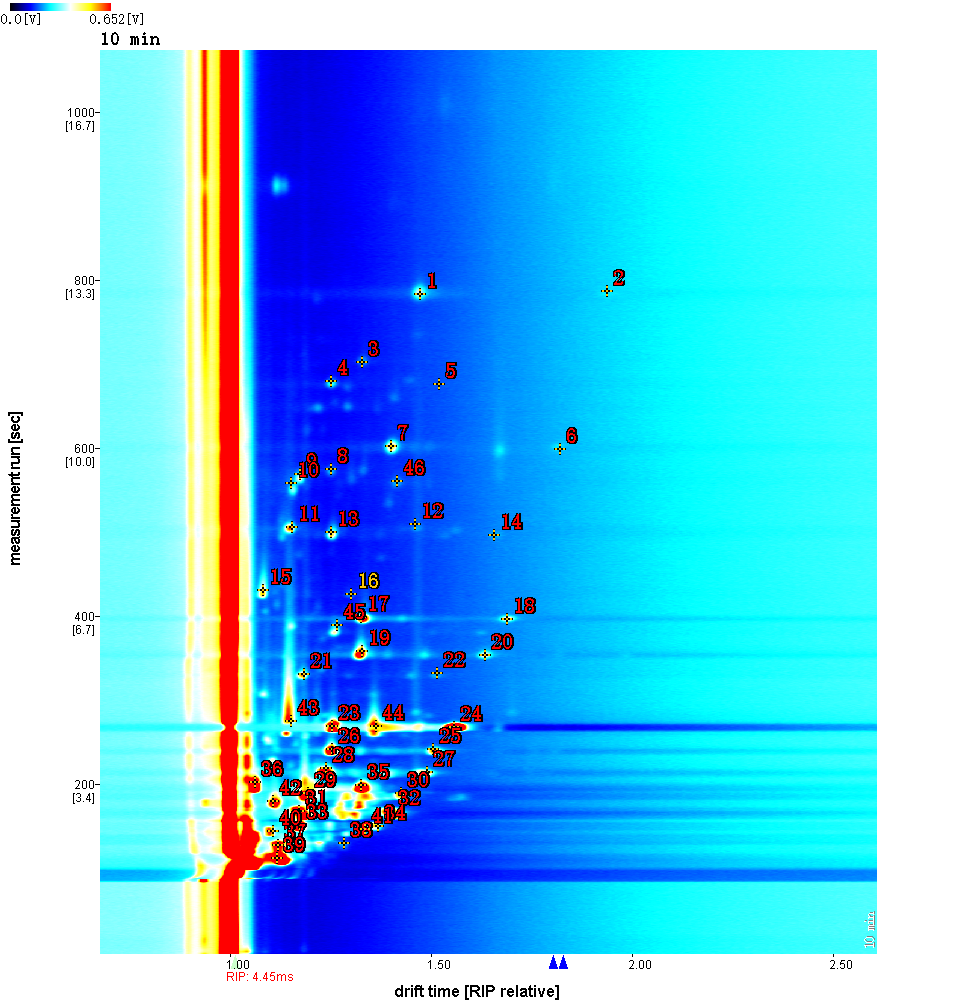

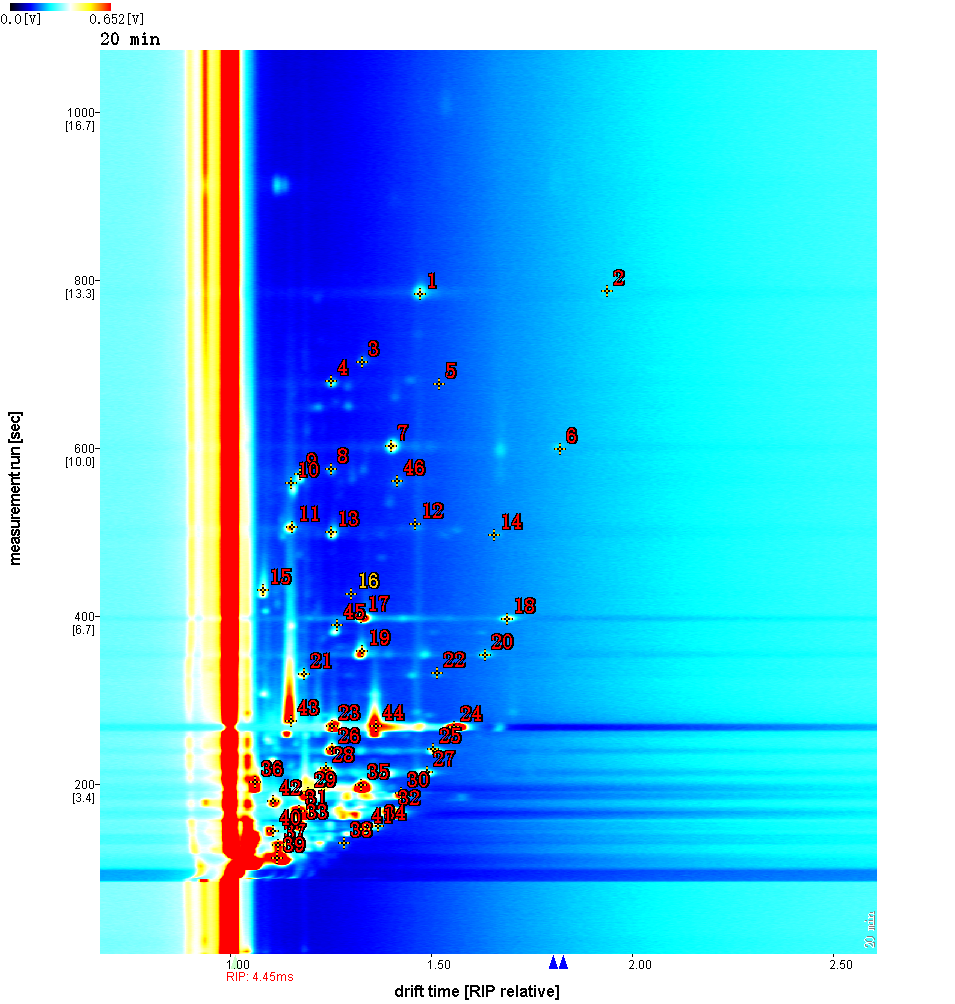

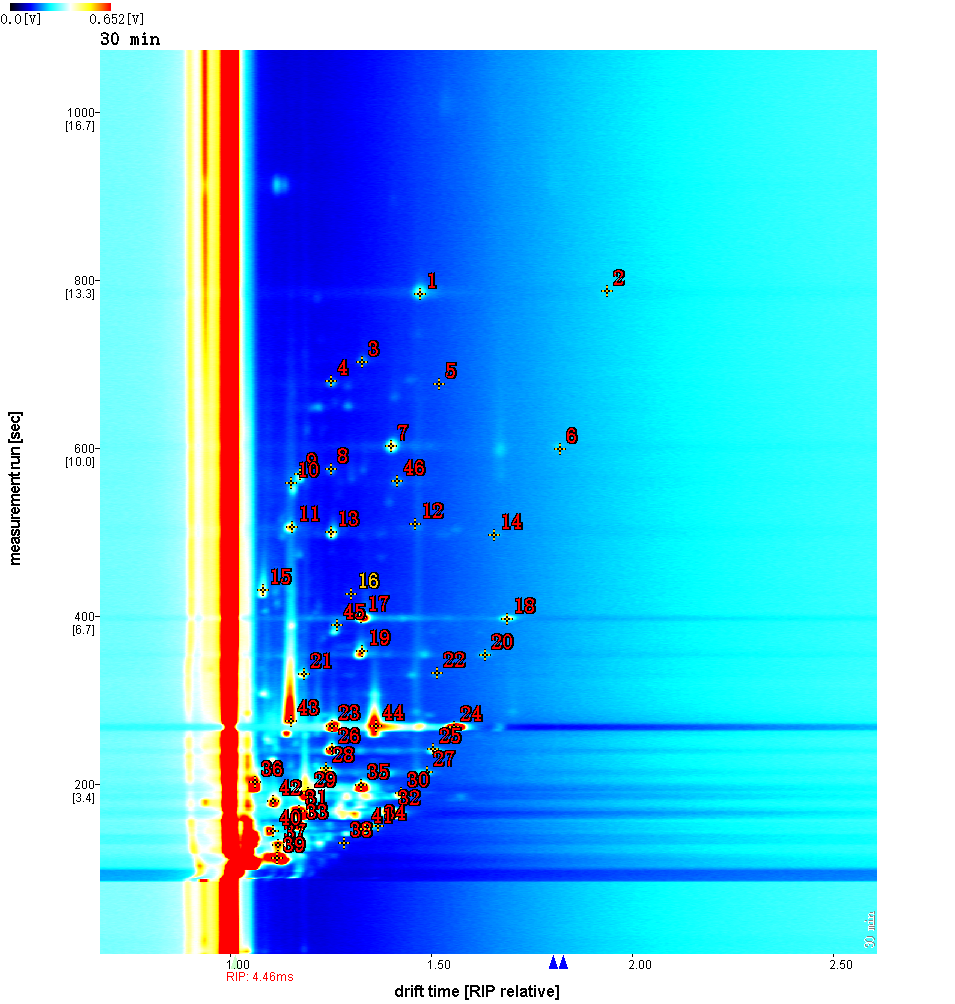


**Figure S1** GC-IMS qualitative profile of volatile components of black rice tea during different roasting times (0, 5, 10, 20, and 30 min)
